# Design for invention: annotation of functional geometry interaction for representing novel working principles

**DOI:** 10.1007/s00163-017-0267-2

**Published:** 2017-09-09

**Authors:** Mark Atherton, Pingfei Jiang, David Harrison, Alessio Malizia

**Affiliations:** 1 Institute of Materials and Manufacturing, Brunel University London, Uxbridge, UB8 3PH UK; 2 College of Engineering, Design and Physical Sciences, Brunel Univesity London, Uxbridge, UB8 3PH UK; 3 School of Creative Arts, University of Hertfordshire, Hatfield, UK

**Keywords:** Function analysis diagram, Functional interactions, Functional representation, Geometric features, Prior art, Semantics, Working principle

## Abstract

In some mechanical engineering devices the novelty or inventive step of a patented design relies heavily upon how geometric features contribute to device functions. Communicating the functional interactions between geometric features in existing patented designs may increase a designer’s awareness of the prior art and thereby avoid conflict with their emerging design. This paper shows how functional representations of geometry interactions can be developed from patent claims to produce novel semantic graphical and text annotations of patent drawings. The approach provides a quick and accurate means for the designer to understand the patent that is well suited to the designer’s natural way of understanding the device. Through several example application cases we show the application of a detailed representation of functional geometry interactions that captures the working principle of familiar mechanical engineering devices described in patents. A computer tool that is being developed to assist the designer to understand prior art is also described.

## Introduction

‘Design intent’ can be defined as “the purpose or underlying rationale behind an object. The intent differs from the functionality in that the intent justifies a design decision whereas the functionality just tells what the design does” (Henderson 1993). It is the core rationale underlying how CAD models and 2D technical drawings should be constructed to communicate functional meaning of a design (Iyer and Mills 2006; Li et al. 2010; Mandorli et al. 2016). The design intent behind the cases in this paper is not known but it is assumed that the designer intended novelty by submitting a patent.

In mechanical engineering, design intent determines the intended relationship between function and the physical arrangement of a device. This design solution can be described as the *working structure* (Pahl and Beitz 1988) that fulfils the overall function of the device being designed. The various sub-functions that contribute to the overall function, herein collectively referred to as functions, are achieved through interrelationships between physical effects (e.g. friction effect, lever ratio, thermal expansion), geometric features (e.g. form, size, location, orientation, surface texture, a screw thread), and material characteristics (e.g. elasticity, coefficient of thermal expansion) known as *working principles* (Pahl and Beitz 1988). For brevity, working principle will be used in place of working structure throughout this paper and the physical effects and material characteristics described can be considered as attributes of geometric features decided by the designer. Therefore, the working principles are achieved through functional interrelationships, or interactions, between geometric features that embody physical effects and material characteristics.

**Fig. 1:**
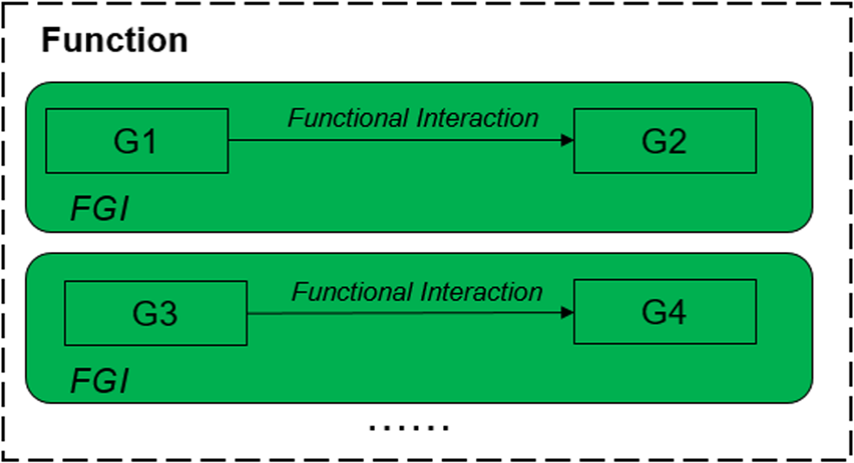
Functional geometry interactions that combine to produce function

We use the term ‘functional geometry interaction (FGI)’ to represent interacting geometrical features (embodying physical effects and material characteristics) that have a functional role in the working principle, e.g. G1 interacts with G2 for a functional purpose intended by the designer (Fig. 1), and several FGI will combine to produce the sub-functions and functions within a working principle.

There is no unified definition of what are geometric features of an engineering design. The international standard for geometrical product specifications (ISO 5459 2011; ISO 14405 2011; ISO 16792 2006) defines a geometrical feature as a point, line or surface and is amplified by B8888 (BS8888 2013) to be the constituent ‘elements’ of a workpiece, whereas others associate them with generic shape entities that carry some engineering meaning (Salomons et al. 1993), and more recently, entities intentionally introduced to satisfy certain requirements (Sanfilippo and Borgo 2016). Identifying geometric features in terms of points, lines and surfaces is insufficient for understanding a working principle. In this paper, the term ‘geometric feature’ is used in the broadest sense to describe a higher level of physical structure that has some functional significance intended by the designer. The term may also be used for a component part, when appropriate or convenient, because for some designs, and certainly some patented designs, the important entity is described as a part because it is the best way to understand it, rather than as many constituent lower level entities. Therefore, for some devices the distinction between a component part and a geometric feature will not be easy to make, as using a part name may be a convenient shorthand for a complex arrangement of primitive entities such as edges and faces, as in the case of a rack and pinion. Whilst the use of the term ‘geometric feature’ would ideally be limited to a coherent unified definition of entities that form a common set across a variety of devices for comparison, our approach is to accept that part names will be a convenient means of making initial progress with the method.

Whilst designers are often just concerned with achieving function using a standard working principle, sometimes their design intent is to create a novel working principle. The novelty of the working principle will be established in relation to the prior art described in patents. In order for a patent to be granted an invention must be novel and involve an inventive step, as well as be capable of industrial application (UK Intellectual Property Office 2014). ‘Novelty’ is simply whether the design is qualitatively different from what has been previously disclosed in the field of application. ‘Inventive step’ is more subjective but is whether the design solution is non-obvious to someone skilled in the art of the field of application. These are both legal definitions best left to patent experts and, therefore, ‘novel working principle’ will be the term used in this paper to indicate the novelty and/or inventive step of a working principle at the time the patent was published.

This paper addresses how to engage designers with patents during their design process rather than afterwards. The primary aim of the paper is to show how graphical functional representations can be associated with mechanical engineering patent drawings/images (and therefore CAD models too) in order to increase awareness of the working principle of the device. For brevity, demonstration of the method is limited to patent examples only, but it can be applied to emerging designs with further development. Communication of the working principle is by means of a novel semantic annotation of patent images combining graphical functional representation with a text summary. It is not intended to describe a legal tool for determining patent infringement but is primarily a design tool to assist the designer to understand prior art and hence, tacitly, avoid patent infringement and promote invention. Therefore, in Sect. 2 we explain the basis of patent infringement in order to be clear that it is a legal judgement, plus the role of patent retrieval and semantic search tools is briefly reviewed in this context. Section 3 introduces functional representation and then explains working principles based on FGI using a simple patented can lid example. Section 4 shows an initial approach to representing FGI in patents through several example cases. A summary text annotation is also demonstrated. The results are discussed in Sect. 5, which also outlines the challenges going forward, followed by concluding comments.

## Background

In this section we will highlight that patent infringement is a significant problem in mechanical engineering, which is a motivation for this research. Whilst patent claims are the key legal instrument for determining infringement in a judicial case, the role of patent drawings and images is vital for designers to understand the working principle of an invention. There are three specific reasons for designers to study prior art (Ulrich and Eppinger 2011):

1. The designer can learn whether an invention infringes existing unexpired patents.
2. Studying the prior art, the designer gets a sense of how similar their invention is to prior inventions and, therefore, how likely they are to be granted a broad patent.
3. The designer develops background knowledge enabling them to craft novel claims.

### Patent infringement and awareness of prior art

From 2012 to 2013 worldwide patent applications grew by 9% to 2.6 million (WIPO 2014), increasing the likelihood that a designer will unwittingly use prior art. Therefore, it is understandable why at least 24% of UK companies experienced an intellectual property (IP) dispute over the past 5 years. Damages were agreed in 30% of cases and averaged £75–£115 k (Weatherall and Webster 2014), highlighting the need for greater designer awareness of potential patent infringement, which can be gained by looking at the prior art.

The basis of a UK patent includes a description of the invention plus drawings and/or CAD model images and one or more claims (UK Intellectual Property Office 2016). The patent claims are the only aspect that define the exclusive right granted to the patent holder but the other aspects support the understanding of the invention. The first claim defines the essential technical features that distinguish the invention from what is already known in the field. A patent is infringed when elements of its claims match elements of the infringing device. In assessing whether a device (or new patent) infringes with patent claims, examiners look to see if the technical features of the device match those described in the patent, primarily as set out in the claims but, importantly, supported by the patent drawings and images.

In mechanical engineering, technical drawings and computer-aided design (CAD) model images are usually pivotal in describing the technical features of a patented invention. These images can also clarify the relationships between features of the patented design, which whilst they may be covered by the claims will not be understood until the images are scrutinised. Linking the patent claims to the patent images through annotation of the images would thus improve the understanding of both and offer an improved means of searching the images.

Patent infringement, novelty and inventive step are primarily legal judgements and, therefore, use of these terms is avoided in this paper, as the method described is primarily a design tool to assist the designer to understand the working principles of prior art and thereby avoid patent infringement and/or promote invention. The conditions in which the designer will avoid conflict with patented prior art are when they are made aware of the working principles in suitably annotated patents using the method described here. The designer will then compare the working principle of their proposed design with the prior art to avoid conflict. This paper focuses on communicating the working principles of patented prior art.

### Semantic search and retrieval of patents

This paper is not concerned with patent search and retrieval *per se* but rather improving awareness of the working principles of prior art through annotation of patents. However, the increasing volume of patents makes searching and analysing them not only more important but also more challenging and hence various tools have been developed. Here we are concerned with patent retrieval for the purpose of comparing patents and in the future for comparing patents with emerging designs of new devices, rather than patent analysis as used to create patent maps, networks and clusters for commercial purposes. Conventionally, in order to identify relevant prior art using a patent retrieval system the designer, or patent professional, will typically enter appropriate keywords and their semantics will have a considerable effect on the results obtained. Therefore, a single search based on the occurrence of several key words rarely captures sufficient prior art and so commercial patent retrieval systems employ text-based search methods augmented by other techniques. For example, natural language processing (NLP) with machine learning (e.g. IBM Watson SIIP platform) has been applied to patent text search, often using statistical inference to enable text search beyond keywords and attach weightings to many different possible search results. However, statistical NLP methods are semantically weak and are only able to predict with acceptable accuracy if given sufficiently large input (Cambria and White 2014). Although the NLP approach to text searching will benefit patent search, this is not the complete picture as the need for image-based approaches is becoming more important as text-based techniques are increasingly problematical (Bhatti and Hanbury 2013). However, content-based image retrieval techniques are not well-suited to patent-images, for example they exploit colour of images whereas patent images are mostly black-and-white. The requirements of a generic patent image retrieval system have been defined (Vrochidis et al. 2010), which includes a semantic-level interpretation of images not present in contemporary patent search systems (Vrochidis et al. 2012; Bhatti and Hanbury 2013). However, semantic search has been limited to the image descriptive text (Abbas et al. 2014) and other patent image retrieval research has focused on image page orientation, segmentation and low-level feature-extraction (e.g. shape) but this does not effectively capture the technical features or working principle of the design (Li et al. 2014).

Document search methods make a distinction between Navigational Search where the aim is to find a particular document, and Research Search, where the aim is to locate a number of documents relating to the search term (Guha et al. 2003). The latter is most relevant to semantic search of patents for the purpose of prior art awareness and by inference avoiding patent infringement. Semantic search is concerned not just with the occurrence of words but also with their meaning in combination with other words. Ontology, defined as specification of a conceptualisation, is frequently used to encode semantics such that they differentiate based on knowledge and relationships external to the documents being searched. External sources such as ontologies containing semantic knowledge increasingly use linked open data (LOD) where structured formal data are expressed in ontology web language. This type of semantic search is also referred to as ontology-based search and two approaches are identified (Bontcheva et al. 2014): First, human-encoded semantics, where a person encodes semantics in a machine-readable format at the time of document publishing. This typically conforms to a standard such as resource description framework (RDF) or schema that are extensions to web mark-up languages for publishing LODs. Whilst this approach is more accurate it also requires considerable human input effort. Second, Automatic Semantic Annotation, where semantics are generated automatically; the advantage being that machine-readable semantics can be generated for all documents including pre-existing but there is a loss of accuracy. Given the importance of avoiding failure to find an important relevant patent then the first approach will be taken here. More can be found on semantic search in (Bontcheva et al. 2014).

It has already been highlighted above that patent images can be searched by low-level features or by using associated text such as titles or descriptions. However, it is important to bear in mind that the visual similarity of images, as searched by patent image retrieval systems such as PATSEEK and PatMedia (Bhatti and Hanbury 2013), is not necessarily the same as similarity of working principle of the designs that the images depict. In other words, a common working principle between designs suggests to the designer that there is a clash with the prior art. This is an especially important distinction in mechanical engineering as explaining the working principle in many mechanical design patents relies heavily on illustrating how functional relationships depend on novel geometric features. Therefore, creating patent image annotations that capture working principle, based on technical features and functional interactions, will enable more accurate patent search and retrieval.

## Functional representation and functional geometry interaction

### Representation of function and form

Functional representations are well-established in engineering (Rodenacker 1966; Roth et al. 1972; Koller 1973; Hubka 1982; Pahl and Beitz 1988) where design activity is viewed as the establishment of functions related to energy, materials and signals, as appropriate. In the design of complex systems, design process follows a general systematic procedure of breaking the system function down into sub-functions, known as function decomposition. A function is both the general transformative input/output relationship of a system performing a task such as heating, measuring and squeezing; and non-transformative operations such as retaining, guiding, sealing and supporting.

Functions have generally been described using uncontrolled (arbitrary) verb-noun couplets such as ‘transfer force’, ‘reduce speed’, ‘retain bearing’, ‘guide tool’, ‘seal gap’ and ‘support beam’. Form-independent methods, that typically represent the function structure only, necessitate switching between function and form-based reasoning, whereas form-dependent methods, that superimpose function structure onto physical structure, more naturally reflect the designers’ reasoning (Aurisicchio et al. 2012, 2013). A controlled vocabulary of functions called reconciled functional basis (RFB) has been broadly applied in form-independent methods (Hirtz et al. 2002). While RFB has received academic criticisms (Aurisicchio et al. 2013) it was decided to incorporate a development of it in this paper since it provides a standard format of functional representation for the purposes of our research. Form in the context of conventional form-dependent representation usually means the structure at the level of components and higher, whereas geometric features that are often the key design detail are a lower level description than the component level and comprise primitives such as edges, holes and surfaces that may combined to form the feature or a structure.

Therefore, in mechanical design the use of semantic annotation (functional representations plus text summaries) can provide insight into how a working principle is actually achieved by the interaction between geometric features.

As a form-dependent functional representation method, the function analysis diagram (FAD) uses blocks to represent device structure and arrows with labels to represent functional relations between components. However, examples of applying conventional FAD (e.g. Aurisicchio et al. 2013) are limited to product component level, whilst its capability in capturing specific novel features of geometry is unclear. FAD originated in Invention Machine Goldfire software (Devoino et al. 1997) based upon TRIZ methodology (Altshuller 1996) and was originally intended to capture the complex network of interconnected functional relationships between subsystems common in process system design, which explains why geometric detail is overlooked.

### Functional geometry interaction example

**Fig. 2:**
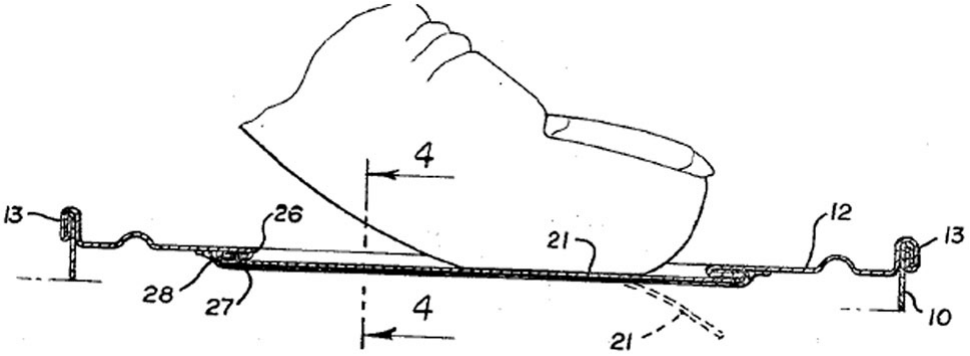
Gated can lid cross-section from 1967 patent (US3334775)

Functional geometry interaction (FGI) can be explained with reference to a simple example: the familiar gated can lid used to seal beverage cans until they are opened by the consumer. This expired patent example and others in the paper are chosen on the basis that they are familiar everyday items with working principles that will be readily understood. They also avoid any current commercial infringement controversy and allow plenty of time for any infringement cases to have appeared in the literature. A cross-section of common gated can lid extracted from a 1967 patent is illustrated in Fig. 2.

**Fig. 3:**
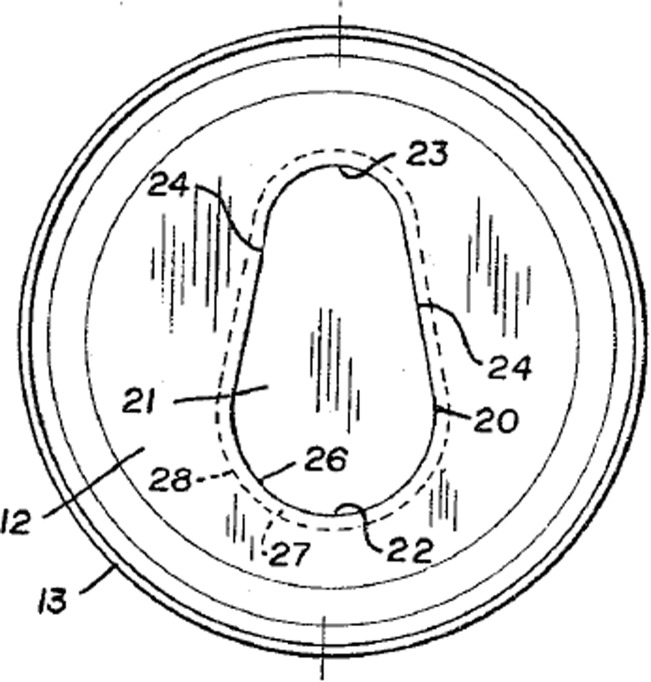
Top of 1967 gated can lid patent (US3334775)

The gate is the panel of the lid (label 21) that becomes an aperture when pushed open due to fracture along a scored line in the lid material (usually aluminium); the aperture perimeter is shown in Fig. 3 (labels 20, 22, 23, 24).

**Fig. 4:**
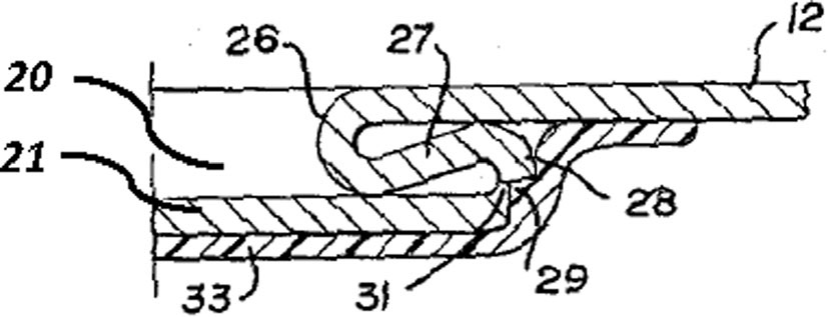
Cross-section detail of 1967 gated can lid patent (US3334775)

Figure 4 shows the geometry of the aperture-forming gated can lid in close detail.

In the rest of the paper only the components of the FGI, namely geometric features and their functional interactions will be highlighted. A Geometric feature specified in patents will be identified by an Underline and a *functional interaction* between geometric features will be shown in *italics*. The underside protective Resin (label 33) can be ignored. The Can lid (label 12) is a single sheet of aluminium that has a Double folded edge (labels 26 and 28) defining the Aperture (label 20) and Gate panel (label 21). This means that the Gate panel (label 21) is underneath and larger than the Aperture (label 20) that will be created when the Score cut (label 29) fails, which protects the consumer from the Sharp edge. Initial fracture of the Score cut (label 29) releases the pressure within the can and after a slight pause in action the consumer will continue tearing the rest of the Gate panel (label 21) from the Can lid (label 12) along the Score cut (label 29) to produce the complete Aperture (label 20). On closer inspection of the patent it is clear how the designer achieved his design intent for the two functions of creating gate-opening and edge separation safety. The function of creating the gate-opening depends upon the functional interaction of *allow separation* between the Score cut (label 29) and the Gate panel (label 21), and the *separate* functional interaction between the Gate panel (label 21) and Spacer strip (label 27) when it is pressed by the consumer.

At the same time, edge separation safety basically depends upon a *surround* functional interaction between the Double-folded edge (labels 26 and 28) and both the Score cut (label 29) and Neck (label 31). The Aperture (label 20) is stiffened by the Double-folded edge (label 26 and 28), which also aids the gate-opening.

In other words, the designer has carefully made complex decisions (their design intent) about the attributes (e.g. physical effects and material characteristics) of these seemingly simple FGI in order to achieve satisfactory functions. For example, if the Neck (label 31) produced by the Score cut (label 29) is too thin then it will prematurely fail under the pressure of the beverage, and if too thick it will be too difficult for the consumer to initiate fracture in order to open the Gate panel (label 21). Similarly, the geometry of the Double-folded edge (label 26 and 28) has to be chosen by the designer to have proximity to the finger for transferring cleaving force for fracture initiation that is balanced against separation from the finger and lips so that the consumer avoids receiving cuts from the Sharp edge produced by the fracturing and tearing of the Gate panel (label 21) from the Aperture (label 20). Even for this simple example it is clear that there are several quite complex FGI that have been considered by the designer in deciding feature details of the design. These FGI determine the novel working principle, which link to the inventive step or novelty of the invention claimed by the patent.

## Representing functional geometry interaction

Designs heavily reliant on FGI for their working principle can be annotated in two steps in order to help the designer understand the prior art. First, a detailed functional representation that highlights the key FGI can be developed from the information on working principle contained in patent independent claims and existing patent images. FGI are identified as key because they come from the independent claims. This graphical annotation representation is embedded in the patent document and may be hidden, available to the designer on request, as it is detailed and, therefore, potentially overwhelming. Second, based on the detailed graphical annotation, a concise text summary is produced outlining the important characteristics of the geometry directly linked to the functional advantage. For example, in the case of the gated can lid, the function create gate opening will normally have been addressed in the descriptive parts of the relevant patent but understanding is enhanced by the FGI representation that comes from the first step.

We will use the 1967 gated can lid design described in Sect. 3.2, together with an earlier 1952 patent for a gated can lid, in order to illustrate how conventional FAD falls short of the detail required to graphically represent the FGI central to their working principles. This will lead onto an improved functional representation that adequately captures FGI.

### Functional representation using conventional FAD

**Fig. 5:**
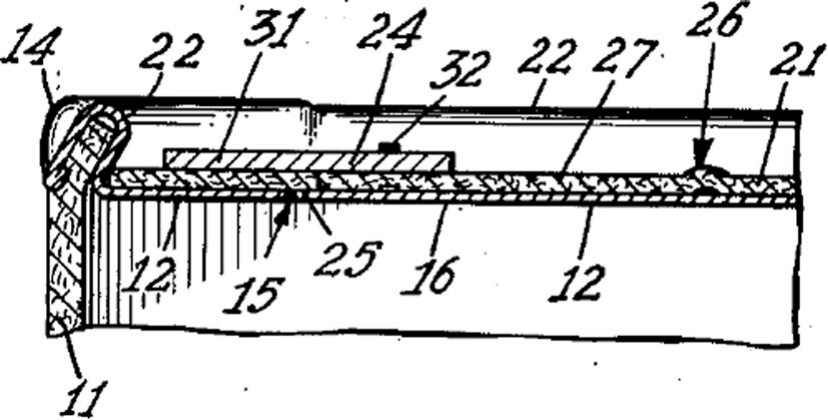
Cross-section detail of 1952 gated can lid patent (US2615610)

Figure 5 shows the cross section detail of a 1952 patented can end design in which a felt Shield (label 21) protects the Raw cutting edge when the Score line (label 15) is broken, and it also serves as a Reclosure element for the can.

**Fig. 6:**
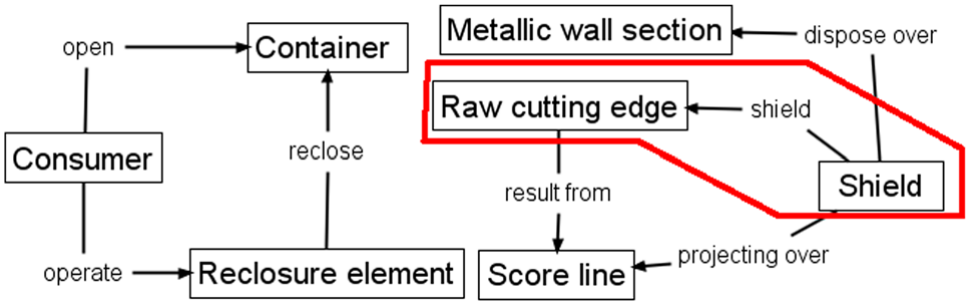
Conventional FAD applied to 1952 gated can lid patent (US2615610)

Figure 6 shows conventional FAD applied to the 1952 patent following the procedure outlined in (Aurisicchio et al. 2012). Features of the invention are represented in the boxes where feature names and functional interactions use the phrases stated in the patent and only useful functional interactions are shown in the figure. Important outside objects that interact with the device, e.g. the consumer, are also represented in a box. The red outline indicates an area of interest for discussion.

**Fig. 7:**
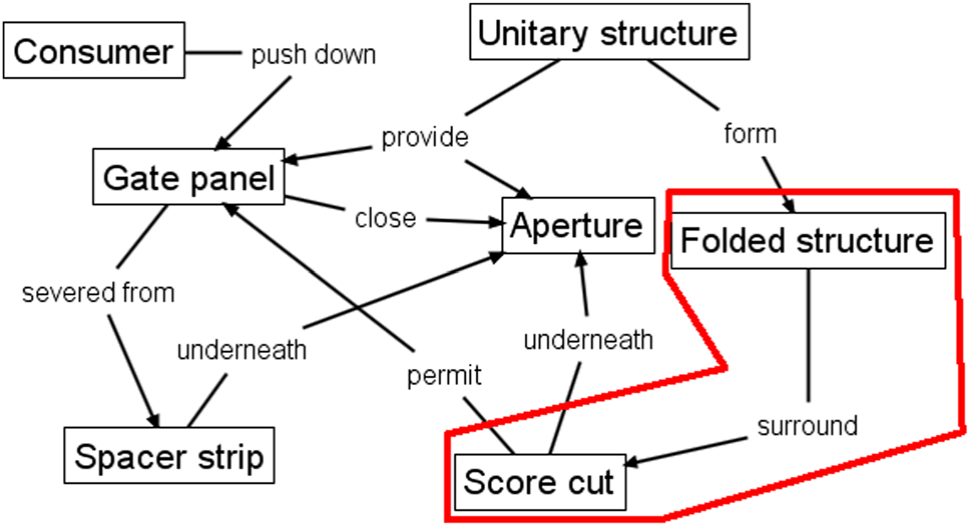
Conventional FAD applied to 1967 gated can lid patent (US3334775)

Figure 7 shows conventional FAD applied to the 1967 patent, described in detail in Sect. 3.2, where the red outline is an area of interest for comparison with that of Fig. 6.

When the elements contained within the red outlined areas of Figs. 6 and 7 are compared, it reveals that both designs have a functional interaction, *shield* and *surround,* respectively, between the Raw cutting edge/Score cut, respectively, and another design feature (Shield/Folded structure, respectively) that fulfils the function of edge separation safety. This comparison implies that there is potential similarity in relation to how each design provides the edge separation safety function by isolating the Sharp edge from the consumer created by breaking the Score cut. However, the fact that there is no clear conflict with prior art is indicated by direct comparison of the patent independent claims for the two cases. In Table 1 there is a comparison of the geometric features extracted from the patent independent claims (which will be found later in Figs. 9 and 11) showing that they do not conflict. Also, there is no record in the literature of any legal cases raised regarding infringement between these two cases.

**Table 1:** Comparison of geometric features in 1952 and 1967 gated can lid patents

US2615610 (1952) claim elements (geometric features only)	US3334775 (1967) claim elements (geometric features only)
Container	
	Unitary structure
Metallic wall section	Gated can lid
Dispensing opening	
Depressible area	Gate panel
Opening	Aperture
Raw cutting edge	Gate panel edge
Score line	Score cut
Shield	
Reclosure element	
	Aperture edge
	Flat sheet metal can lid member
	Inward underfold
	Outward underfold
	Spacer strip
	Spacer strip outer edge

Similar geometric features are aligned to visualise comparison

Therefore, it can be seen that conventional FAD is unable to satisfactorily distinguish between the two designs (Figs. 6, 7) because it does not represent sufficient detail to avoid jumping to the wrong conclusion that the newer patent conflicts with the older patent based on the similarity of functional relationships between the key components. Consequently, FAD Plus, or FAD+, has been developed to capture working principles at a more detailed level through better representation of functional geometry interaction. It is at this level of detail that conflict of prior art can be shown to occur in mechanical engineering design, as follows.

### Representing functional geometry interaction using FAD plus (FAD+)

FAD+ enhances the diagrammatic representation of mechanical inventions beyond FAD in terms of key detailed geometric features described in patent claims and images and also represents invention hierarchy. In addition, FAD+ uses functional interaction terms developed from RFB.

**Fig. 8:**
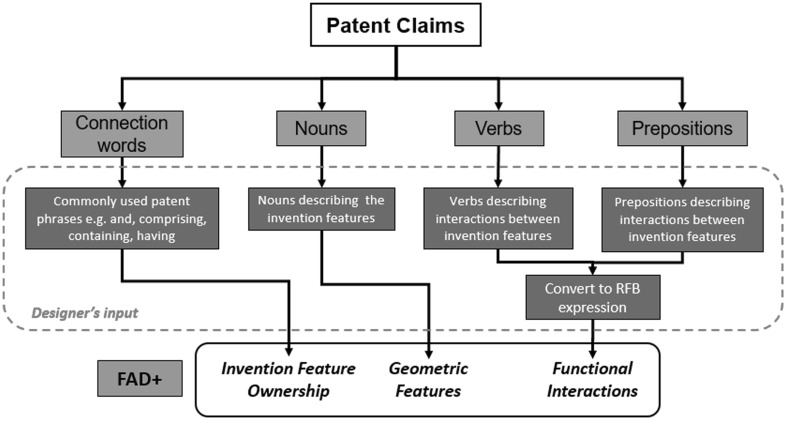
Process for generating FAD+ diagram with designer’s input

Information required for developing FAD+ can be gathered from words and phrases contained within patent claims that can be categorised as geometric features and functional interactions. For example, nouns describing the invention features can be classified as geometric features and verbs can be classified as functional interactions between geometric features. These terms will be expressed using RFB for the purpose of conceptualisation and standardisation. Below is demonstrated how FAD+ diagrams were produced for the two gated can patent examples. For simplicity, only the independent claim was used and the process for generating the FAD+ diagram with the designer’s input is illustrated in Fig. 8.

#### Gated can lid examples of applying FAD+

**Fig. 9:**
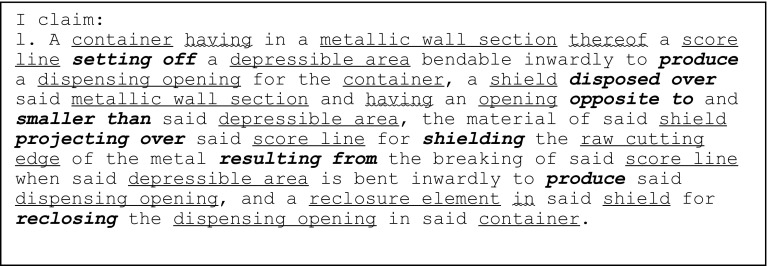
Independent claim of the 1952 gated can lid patent (US2615610)

The independent claim of the 1952 gated can lid patent (US2615610) is shown in Fig. 9 with key geometric features underlined, functional interactions in bold italics and feature ownership identified by a wavy underline.

Information gathered at each stage of FAD+ is also presented to provide visibility of knowledge extraction. Tables 2, 3 and 4 summarise the geometric features, feature ownership and FGI developed from the patent document.

**Table 2:** Geometric features identified in independent claim of US2615610

Geometric features
Container
Depressible area
Dispensing opening
Metallic wall section
Opening
Raw cutting edge
Reclosure element
Score line
Shield

**Table 3:** Feature ownership identified in independent claim of US2615610

Feature ownership		
Geometric features	Ownership	Geometric features
Container	having	Metallic wall section
Container	, and	Shield
Metallic wall section	in….thereof	Score line
Shield	having	Opening
Reclosure element	in	Shield

**Table 4:** FGI developed from independent claim of US2615610

Working principle				
	Geometric feature #1	Patent functional interaction term	Functional interaction RFB expression	Geometric feature #2
FGI #1	Score line	Setting off	Provide	Depressible area
FGI #2	Depressible area	Produce	Generate	Dispensing opening
FGI #3	Shield	Dispose over	Locate above	Metallic wall section
FGI #4	Opening	Opposite to	Locate opposite	Depressible area
FGI #5	Opening	Smaller than	Smaller	Depressible area
FGI #6	Shield	Projecting over	Extend over	Score line
FGI #7	Shield	Shielding	Cover	Raw cutting edge
FGI #8	Score line	Result	Provide	Raw cutting edge
FGI #9	Reclosure element	Reclosing	Reclose	Dispensing opening

**Fig. 10:**
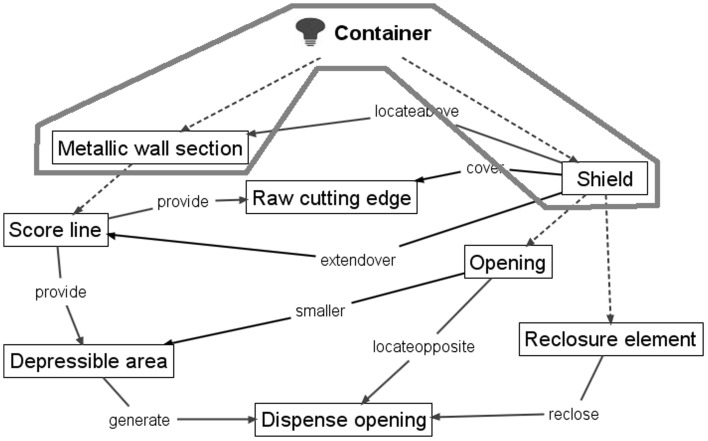
FAD+ for 1952 gated can lid patent (US2615610)

Applying FAD+ to the two gated can lid patents described previously in Figs. 4 and 5 using the approach described in Fig. 8, the FAD+ graphical representation is shown in Figs. 10 and 12. Feature ownership between geometric features are shown in dashed lines and functional interactions are shown in solid lines. The red outline indicates an area of interest for discussion later.

**Fig. 11:**
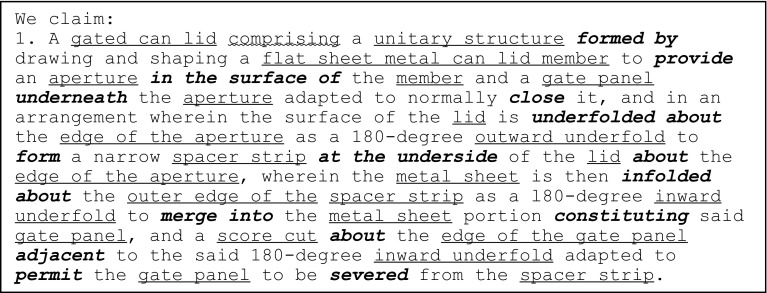
Independent claim of the 1967 gated can lid patent (US3334775)

Similarly, the independent claim of the 1967 gated can lid patent (US3334775) is shown in Fig. 11 with key geometric features underlined, functional interactions in bold italics and feature ownership identified by a wavy underline.

Again, information gathered at each stage of FAD+ is also presented in Tables 5, 6 and 7, which summarise the geometric features, feature ownership and FGI developed from the patent document.

**Table 5:** Geometric features identified in independent claim of US3334775

Geometric features
Gated can lid
Aperture
Aperture edge
Flat sheet metal can lid member
Gate panel
Gate panel edge
Inward underfold
Outward underfold
Score cut
Spacer strip
Spacer strip outer edge
Unitary structure

**Table 6:** Feature ownership identified in independent claim of US3334775

Feature ownership		
Geometric features	Ownership	Geometric features
Gated can lid	Comprising	Unitary structure

**Table 7:** FGI developed from independent claim of US3334775

Working principle				
	Geometric feature #1	Patent functional interaction term	Functional interaction RFB expression	Geometric feature #2
FGI #1	Flat sheet metal can lid member	Form	Form	Unitary structure
FGI #2	Flat sheet metal can lid member	Provide	Provide	Aperture
FGI #3	Aperture	In the surface of	Locate on	Flat sheet metal can lid member
FGI #4	Flat sheet metal can lid member	Provide	Provide	Gate panel
FGI #5	Gate panel	Underneath	Locate under	Aperture
FGI #6	Gate panel	Close	Close	Aperture
FGI #7	Flat sheet metal can lid member	Underfolded about	Surround	Aperture edge
FGI #8	Flat sheet metal can lid member	As	Form	Outward underfold
FGI #9	Outward underfold	Form	Form	Spacer strip
FGI #10	Spacer strip	At the underside of	Locate under	Flat sheet metal can lid member
FGI #11	Spacer strip	About	Surround	Aperture edge
FGI #12	Flat sheet metal can lid member	Infolded about	Surround	Spacer strip outer edge
FGI #13	Flat sheet metal can lid member	As	Dorm	Inward underfold
FGI #14	Inward underfold	Merge into	Merge	Flat sheet metal can lid member
FGI #15	Flat sheet metal can lid member	Constituting	Provide	Gate panel
FGI #16	Score cut	About	Surround	Gate panel edge
FGI #17	Score cut	Adjacent to	Locate adjacent	Inward underfold
FGI #18	Score cut	Permit	Allowseparation	Gate panel
FGI #19	Gate panel	Severed from	Separate	Spacer strip

**Fig. 12:**
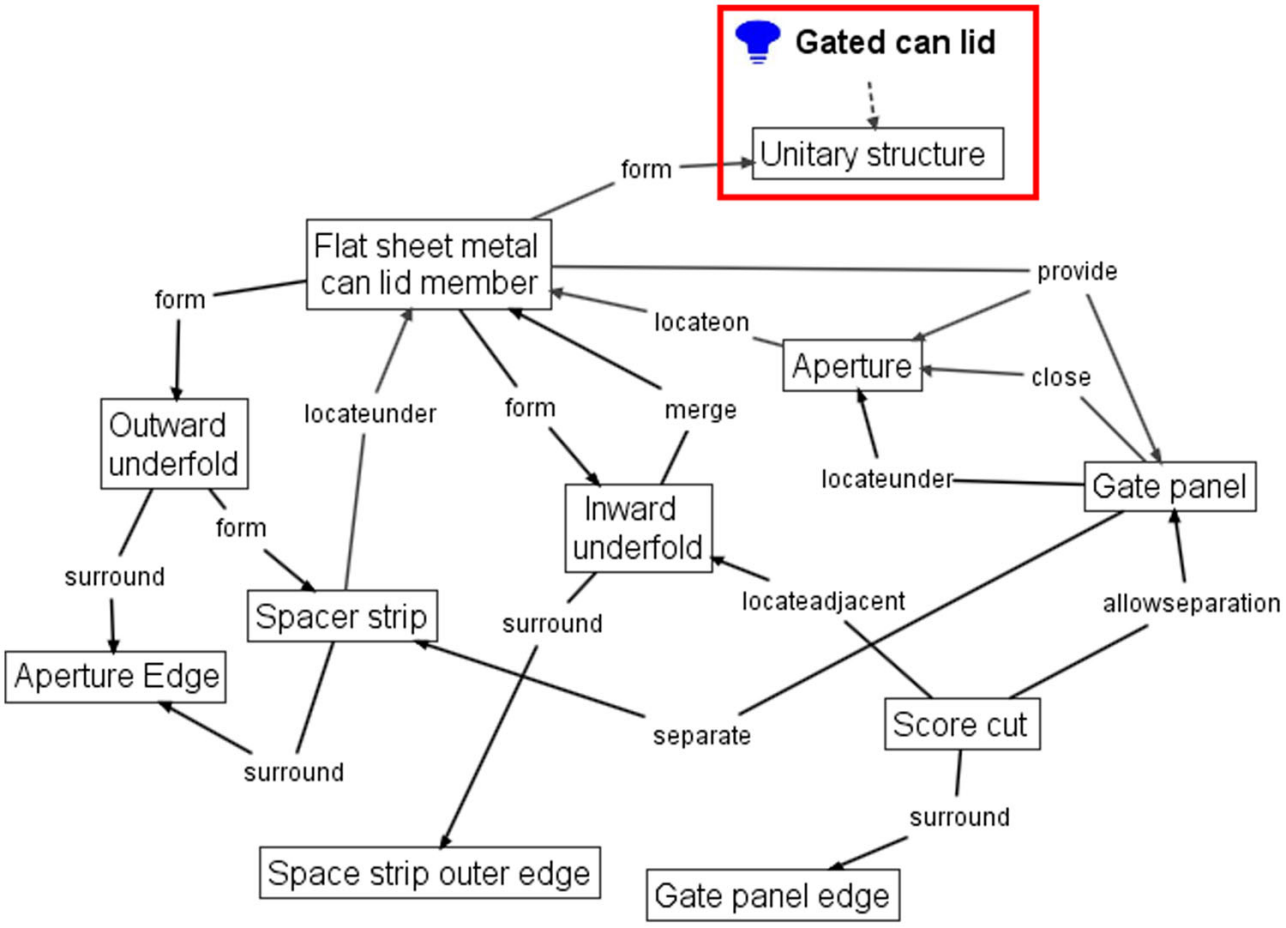
FAD+ for 1967 gated can lid patent (US3334775)

The FAD+ graphical representation using the approach described in Fig. 8 is shown in Fig. 12.

The novel working principle of the design in Fig. 12 centres on how the edge separation safety function was achieved compared to the 1952 gated can lid patent (US2615610). The Gate panel is located under the Aperture, while simultaneously the Aperture edge is surrounded by the Spacer strip formed by the Outward underfold. At the same time the Spacer strip is surrounded by the Inward underfold. These FGI together contribute to the edge separation safety function that protects the consumer from the sharp Gate panel edge created by the Score cut on the Gate panel.

The FAD+ graphical representation derived from the patent independent claims distinguishes between the two gated lid designs more clearly, demonstrated by the FGI. The 1952 patent uses a separate felt Shield which has a smaller Opening than the Aperture, hence the overhang protecting the consumer’s finger from Sharp edge when the Depressible area is fractured along its Score line. However, the 1967 design achieves the same function by means of the Double-folded edge (Inward underfold, Spacer strip and Outward underfold) of a single part. On close inspection of Fig. 12, the novelty of the 1967 patent will be seen to reflect the fact that the Sharp edge of the Gate panel and Double folded edge belong to the same part rather than two separate parts according to the feature ownership shown (highlighted by the dashed line outlined in red in Figs. 10 and 12, respectively).

#### Corkscrew examples of applying FAD+

**Fig. 13:**
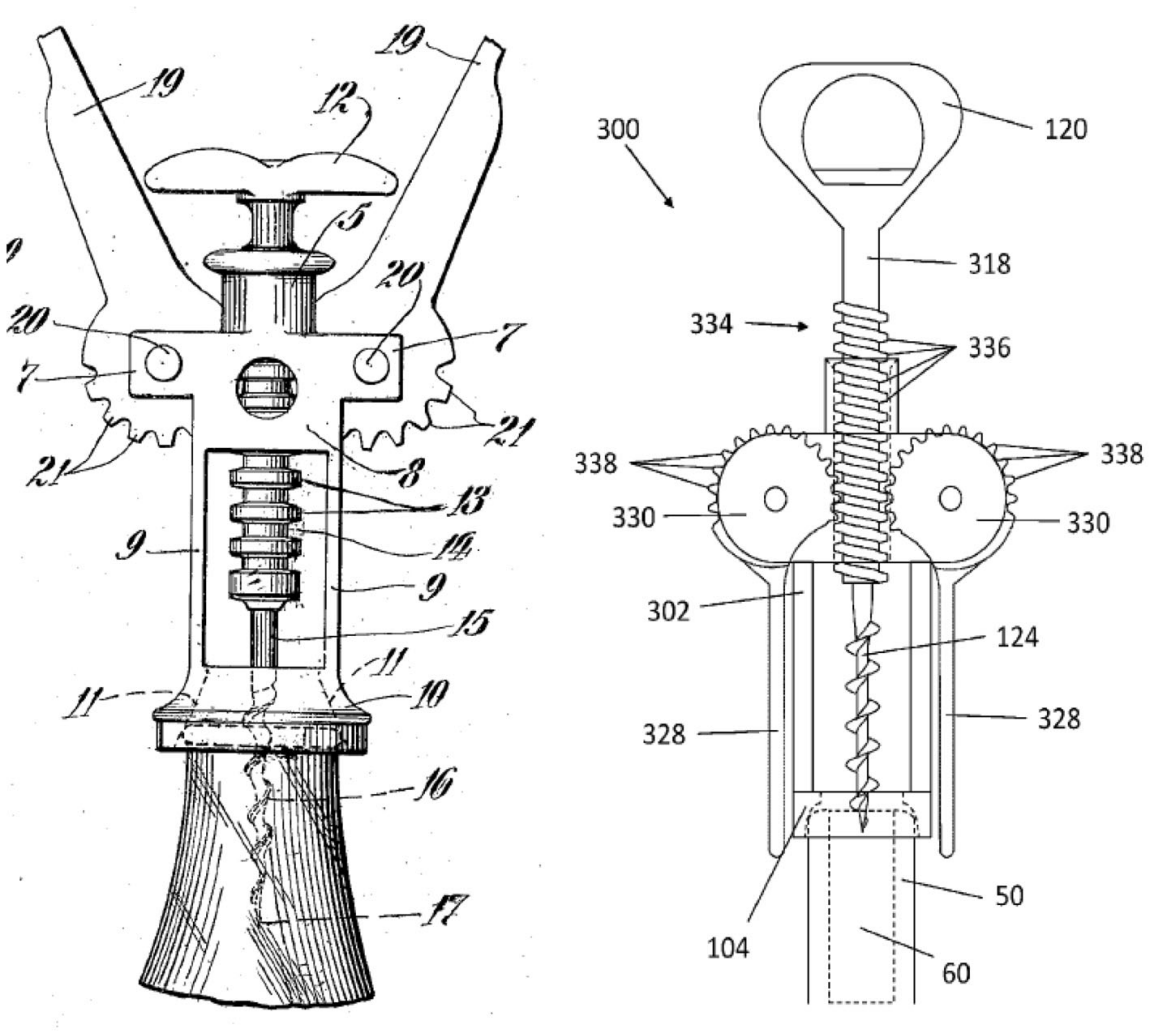
‘Winged’ corkscrew patents: 1930 (*L*) and 2015 (*R*)

Figure 13 shows images from a 2015 corkscrew patent (US 20150191336 A1) (Fig. 13 R) that is a development of the more familiar 1930 “Wing” design (US patent 1753026) (Fig. 13 L) that has been commercially available for a long time and is shown for reference only.

**Fig. 14:**
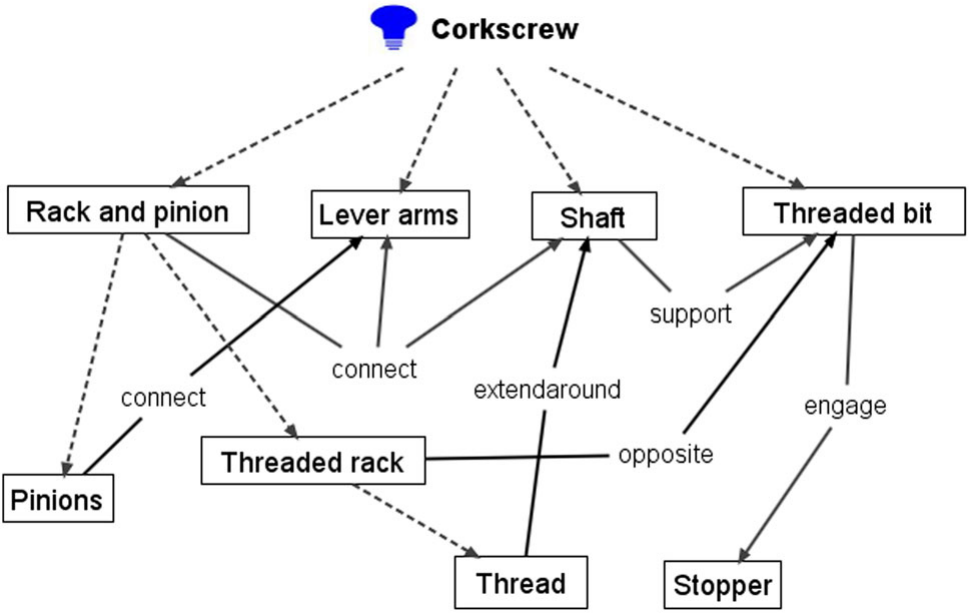
FAD+ for 2015 corkscrew patent (US 20150191336 A1)

Figure 14 shows the result of applying FAD+ to the 2015 corkscrew design omitting the detailed steps demonstrated in the previous gated can lid examples.

The FGI identified originate from the patent independent claims and relate to a departure from a familiar design by using the *opposite* of the Threaded rack (label 334) to interact with the Threaded bit (label 124) in order to amplify the degree of travel of the Lever arms (label 328) for the setting process and shorten the Lever arm (label 328) travel for the removal process enabling one-shot removal of the Stopper (label 60). The 1930 “Wing” design (US patent 1753026) on which it is based has a simple Ribbed rack instead of a Threaded rack.

**Fig. 15:**
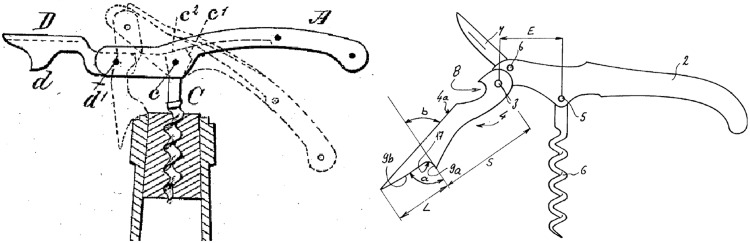
‘Waiter’s friend’ corkscrew patents: 1883 (*L*) and 2002 (*R*)

**Fig. 16:**
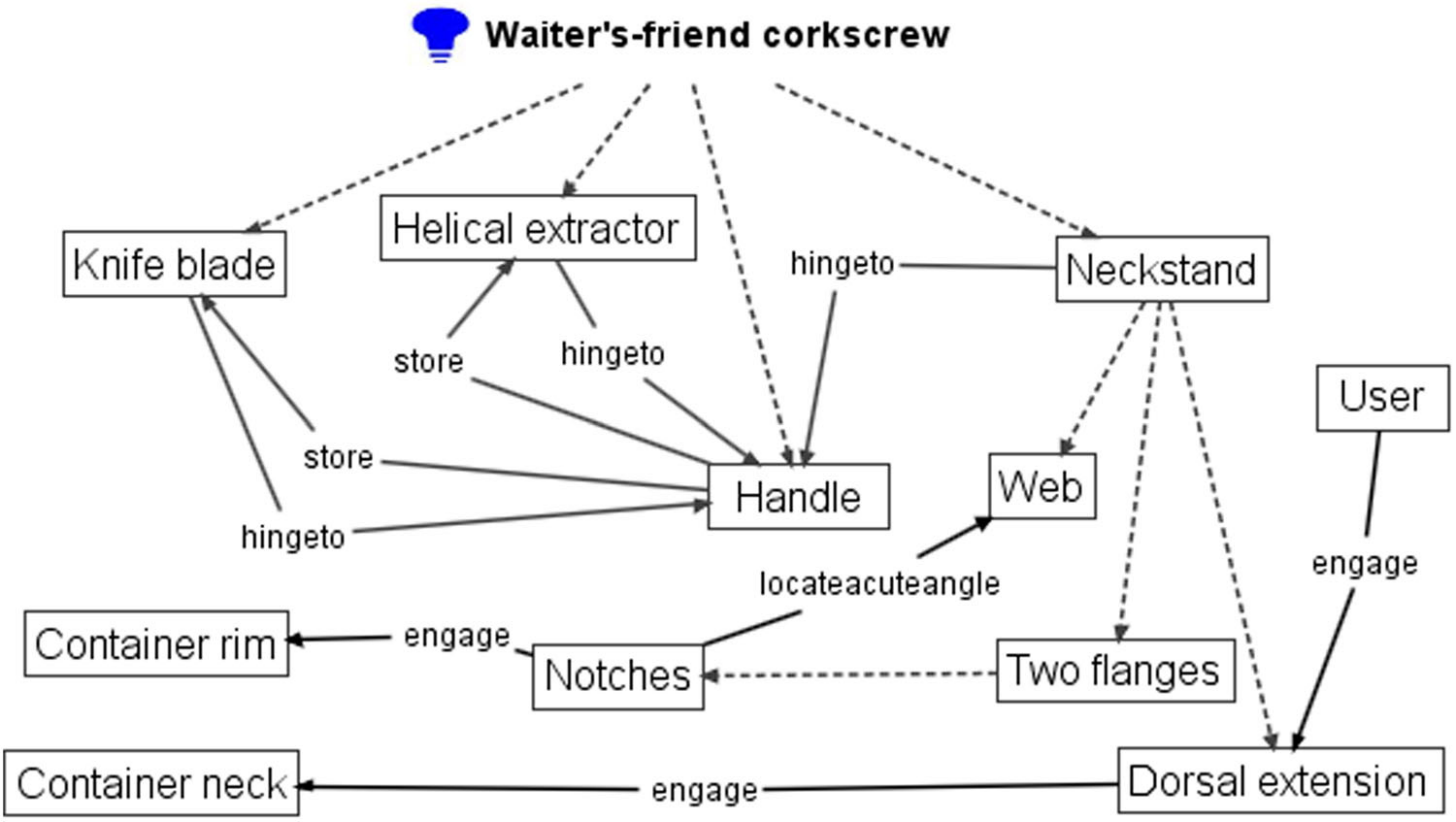
FAD+ for 2002 waiter’s friend corkscrew patent (US2002/0157188 A1)

**Fig. 17:**
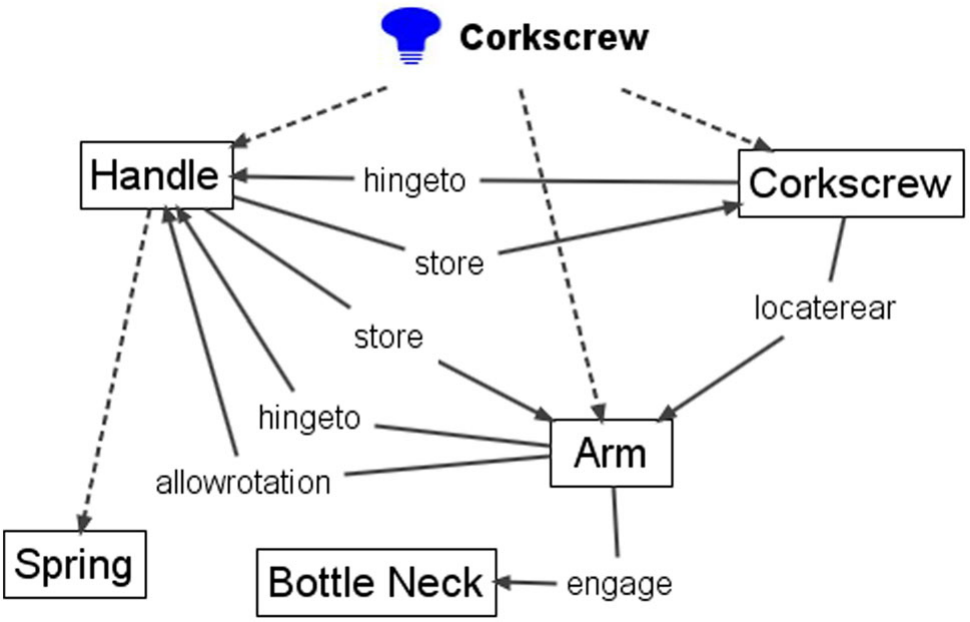
FAD+ for 1883 waiter’s friend corkscrew patent (US283731)

Figure 15 shows two designs of another type of corkscrew, a recent 2002 patent (US20020157188 A1) that is a development of the original 1883 ‘Waiter’s friend’ corkscrew patent (US283731), which is also shown (Figs. 16, 17).

The first novel working principle identified from FAD+ is that the 2002 patent offered another component which is a Knife blade (label 7) *hinged to* and can be *stored* by the Handle (label 2), which will not be considered further. The functional interaction *hingeto* and *store* between Handle (label 2) and Corkscrew (label 6, termed Helical extractor in 2002 patent) are identical in both patents suggesting no novel working principle. However, the 2002 patent offers a more novel working principle based on Dorsal extension (label L) enabling the user’s hand to maintain *engage* Container neck (Bottle neck in 1883 patent) whilst Two flanges (label 9a and 9b) form a Notch (equivalent to 17) to *engage* Container rim. The advantage of this function is that the Neckstand is conveniently brought to bear upon the Container rim by the downward action of using the handle; and the appropriate contact is maintained by the Dorsal extension. The FAD+ has enabled the designer to gain insight into the prior art described by the patent claims and images through revealing the novel working principle represented by the key FGI.

From these case studies it can be seen that FAD+ is concerned with novel geometric details of an invention across a range of mechanical engineering applications. As a result, when analysing complex designs, a product breakdown is suggested as a starting point in order to identify sub-systems and components. FAD+ can be then applied within those sub-systems and components in order to highlight their novel working principles by identifying key functional interactions between the geometric features revealed.

### Text annotation of novel working principle based on FAD+

**Fig. 18:**
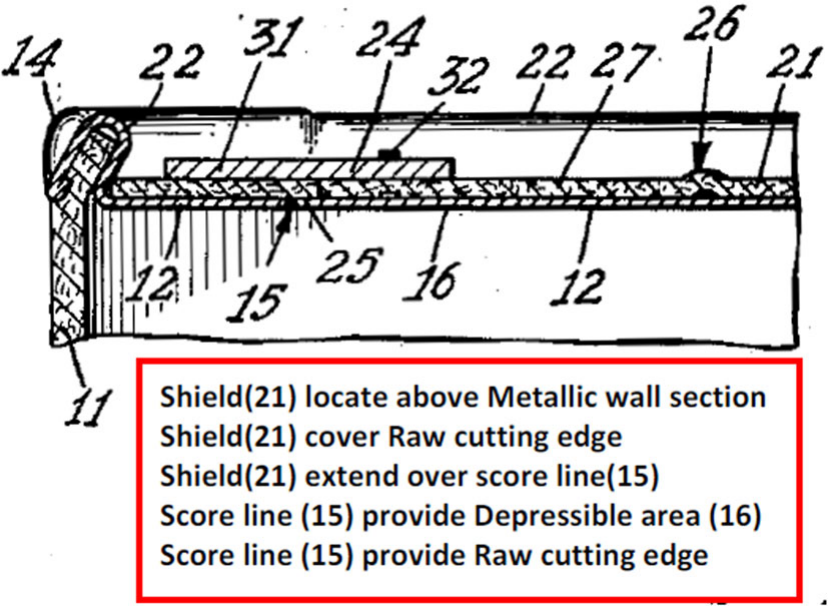
Annotated image of 1952 gated can lid patent (US2615610)

**Fig. 19:**
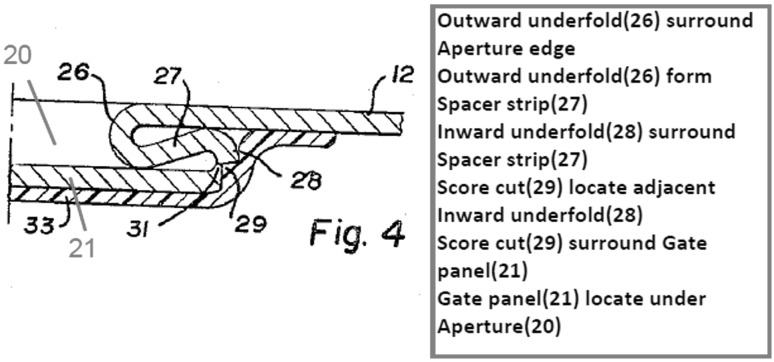
Annotated image of 1967 gated can lid patent (US3334775)

Considering that FAD+ might be too complex to initially present to a designer in a patent image then a text annotation, intended to be read quickly by the designer, can be used as an initial summary of the key FGI that are detailed in a hidden underlying FAD+. The patent images chosen for text annotation would most likely be those most referenced in the patent document. The text summary is generated by collecting the most referenced geometric features as presented in the FAD+ and then including their associated functional interactions and geometric features. Simple phrases are then used. As some of the patent images do not show all of the feature labels needed for the summary, additional labels are added. For example, shield opening (label 56) in Fig. 18, aperture (label 20) and gate panel (label 21) in Fig. 19.

Figure 18 shows text annotation of the 1952 gated can lid patent image highlighting the key geometric features referred to in the short summary of the working principle based on the FAD+. Figure 19 shows the annotated image of the newer 1967 gated can lid patent.

**Fig. 20:**
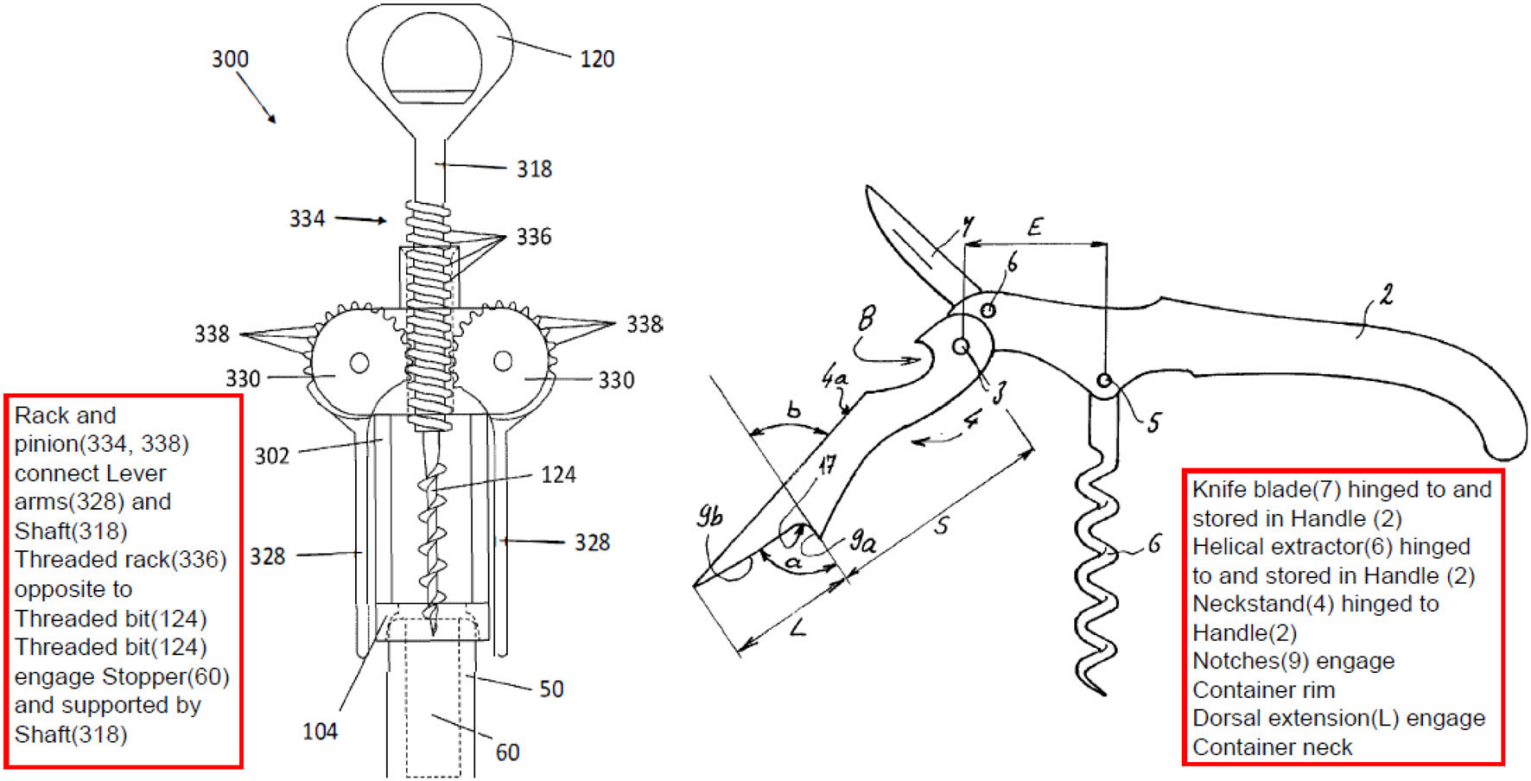
Annotated corkscrew patents (US20150191336 A1 and US2002/0157188 A1)

Figure 20 shows text annotation of the two newer corkscrew patents summarising their novel working principle from the underlying (hidden) FAD+.

A designer will be able to readily understand the novel working principle of each patent with the aid of these annotated patent images derived from FAD+. In practice, rather than occlude the annotated patent figure, FAD+ would initially be hidden then revealed to the designer on request similar to comments revealed by ‘hovering’ over a comment symbol in current PDF documents.

## Discussion

### Developing FAD+ as a graphical patent image annotation tool

The FAD+ method for graphical representation of FGI proposed in this paper is not intended to be a patent tool that confirms inventive step or novelty, rather it aims to highlight the novel working principles and increase the designer’s awareness of relevant patent prior art and thereby avoid patent infringement for their own design.

The method described brings functional modelling into the context of comparing working principles by virtue of the graphical nodes and edges of FAD+, which enables new ways of making statistical comparisons. FAD+ also enables a rigorous transformation of unstructured natural language patent text to structured graphical representation due to the use of ontology, which will enable automated comparison in the future. Our premise is that patent novelty and inventive step as captured by patent claims, description and images relate to details of working principles that can be embodied in FGI for some mechanical engineering designs. Our main focus is on gated can lid design but we have shown relevance to other types of mechanical devices. The gated can lid designs that were compared share the same high-level working principle using FAD and the novelty of the newer design was clearly shown to be in the novel working principle of the geometric detail based on key FGI revealed by FAD+.

Representing some mechanical engineering designs in sufficient detail of geometric features and their interactions is not addressed by FAD, as its application has been limited to the component level. Developing FAD+ to capture lower-level geometric features enables it to represent the working principles of certain classes of mechanical engineering devices as indicated by the example cases. Representing the detailed feature ownership (by the use of dashed lines) is also a novel feature of FAD+ that enhances understanding of working principles. Semantic annotation of patent images summarising the working principle through combined FAD+ and text summaries offers a tangible opportunity for a new patent search and retrieval approach that could provide more accurate results. To be clear, only patents that are annotated in the way described can be searched, which will require a strategic post hoc approach for existing patents. The annotation described in this paper is focused on how to communicate existing patented solutions to the designer and thereby effectively encourage the designer not to use the working principles of those prior art solutions. We see this encouragement to think beyond prior art as the opposite to design fixation (Jansson and Smith 1991).

It has been suggested that FAD needs a better developed syntax in order to be consistent and reliable (Aurisicchio et al. 2013). Our use of domain-specific ontologies in FAD+ addresses this as discussed in the next subsection. However, IHS Goldfire software (Goldfire Technical Knowledge Discovery 2017) employs a semantic indexing technology where high-level semantic subject-action-object items are identified in a sentence for search and trend analysis purposes (Verbitsky 2004) but this is not employed at the level of FGI detail described in this paper. FAD has been shown to have value in analysing complex designs (Lee et al. 2013; Michalakoudis et al. 2014), therefore FAD+ as a simple extension of FAD, should be capable of representing complex designs. Table 8 summarises some main features of FAD and FAD+ for comparison.

**Table 8:** Comparison between FAD and FAD+

FAD	FAD+
Uses natural language No systematic vocabulary Limited to product component level Represents harmful and useful functions	Use standardised vocabulary enabling comparison between designs Provides invention feature ownership Represents geometry detail of component level Adopts systematic approach Potential to be automated

We expect the level of expertise required for FAD+ to be performed is that of a mechanical engineering graduate level of design expertise with at least 2–5 years of professional experience in order to achieve proper understanding of an invention in the domains considered here. The FAD+ diagrams in the above examples took less than 15 min to identify manually from the patent independent claims and generating the text summary took less than 5 min. Adding the graphic and text annotations to the patent document using Adobe Acrobat took less than 3 min. We believe that a FAD or a FAD+ diagram will not differ significantly between professional mechanical design engineers and not differ significantly between specialists in the device domain. However, there may well be significant differences between results from general design engineers and those from design specialists within a specific field of application. We anticipate that this process would be considerably speeded up by future automation that captures basic information about existing patents from the internet, which will mean that they are then “suggested” to the designer. The designer can then be incentivised by the User Interface of a tool currently being developed (see next section) to add more information to patents of interest, such as generating a FAD+ diagram, which then belongs to his/her patent database (which could be shared). Over time the patent database grows in relation to what has been interesting. Perhaps, third parties such as consultancies and universities will populate and share similar databases. Additionally, generating a FAD+ diagram might aid the process of writing patent claims.

### Design assistant tool concept to highlight patent prior art

**Fig. 21:**
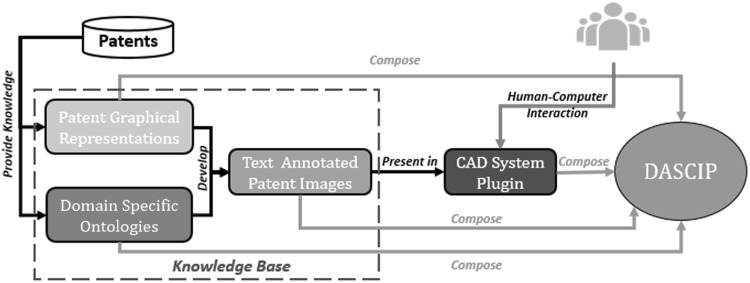
DASCIP overview and core components

A design assistant tool based on FAD+ is being developed that will aim to identify potential commonality of working principle between an emerging design and existing patents and hence identify conflict with prior art that could lead to *potential* infringement. Therefore, design effort will be steered away from patent conflict and towards novelty by providing real-time feedback to the designer. The sets of patents to be used will be drawn from can design, and other domains are yet to be identified. The tool envisaged, identified as Design Assistant for Semantic Comparison of Intellectual Property or DASCIP (Fig. 21), will store FAD+ diagrams and text annotations of the emerging design (3D model or patent image) that express the working principle in terms of descriptions of the FGI using domain-specific knowledge base developed for this purpose.

Figure 21 illustrates an overview of DASCIP and its core components: domain-specific ontologies, patent graphical representation, text annotated patent images and a CAD system plugin.

- *Domain-specific ontologies* This component contains conceptualisation of commonly used design features and FGI with the purpose of standardising terms.
- *Patent graphical representation* Graphical representation of a patent (FAD+) captures patent information and FGI between geometric features of an invention and hence provides insight on working principles. This allows the designer to sense the novelty and inventive step of the invention.
- *Text annotated patent images* Annotated patent images offer a simple way to allow the designer to access the patent information and quickly obtain its core working principle.
- *CAD system plugin* This component enables human–computer interaction to conduct emerging design FAD+ construction and perform comparison to patents.

Initially, it is envisaged that a database of patents will be accessed by conventional search. This database of patents will be used to provide information to develop patent graphical representation and domain-specific ontologies. Annotated patent images will be developed upon successful completion of these two stages and become a new annotated patent database that can be subject to new search techniques to be developed.

**Fig. 22:**
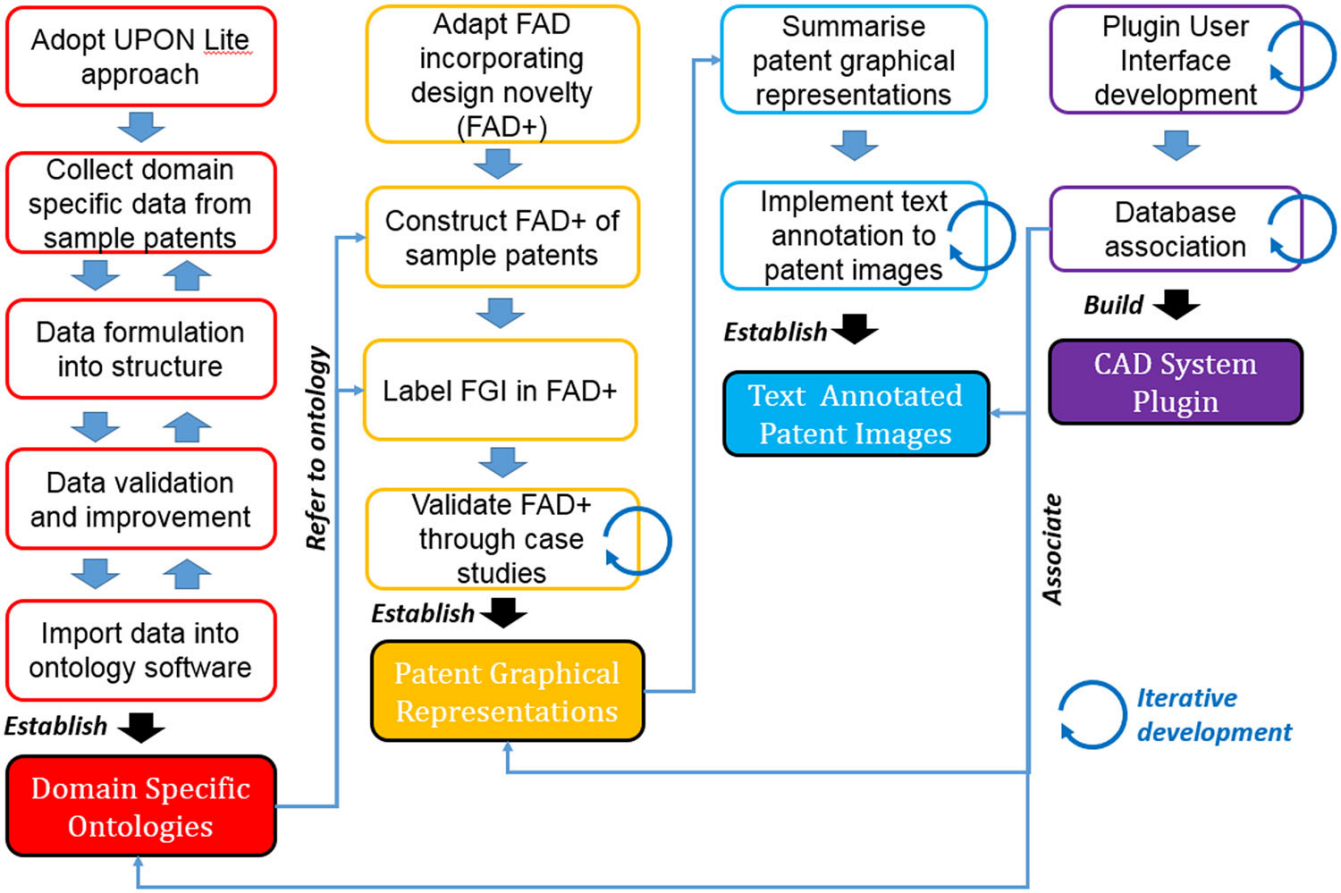
DASCIP development process

Patent graphical representation, domain-specific ontologies and annotated patent images form a knowledge base which contains necessary data to perform FGI interaction analysis and similar prior art identification. Details regarding development of DASCIP core components are shown in Fig. 22.

Domain-specific ontologies will be developed adopting the UPON Lite Ontology Engineering approach (De Nicola and Missikoff 2016). The database of patents will be analysed in order to collect domain-specific data. The data will be then formulated into spreadsheets, validated and improved by domain experts (Jiang et al. 2017). Then structured data will be imported into ontology software to perform contradiction analysis and iterative improvement. At the end of this stage computerised ontologies should be ready to use.

FAD+ will be used to develop a graphical patent representation. It will incorporate the capability of representing invention hierarchy, invention geometric features and functional interactions. Terms defined in domain-specific ontologies will be employed to describe the FGI of patents. FAD+ will be validated and improved iteratively through a number of case studies and then be employed to establish a database of patent functional models with FGI identified.

Patent graphical representation will be summarised by simple sentences containing key invention features and FGI in order to develop the text annotations. At the end of this stage a database of annotated patent images will be established.

Semantic annotations will be linked to an external annotated patent database using XML files (see Camba et al. 2014 for method). The patents will be searched for comparison with the emerging design based primarily on text annotations of the original patent images plus, where relevant, a graphical representation of the design depicted in the patent. Differences in the words used in annotations can be mitigated by a reference ontology mapped to appropriate terms for working principles. A statistical analysis will be performed on the degree of association identified between aspects of the emerging design and relevant patents. Effective ways of visualising the results will be explored, not limited to statistical summaries but perhaps highlighting portions of the relevant patents.

**Fig. 23:**
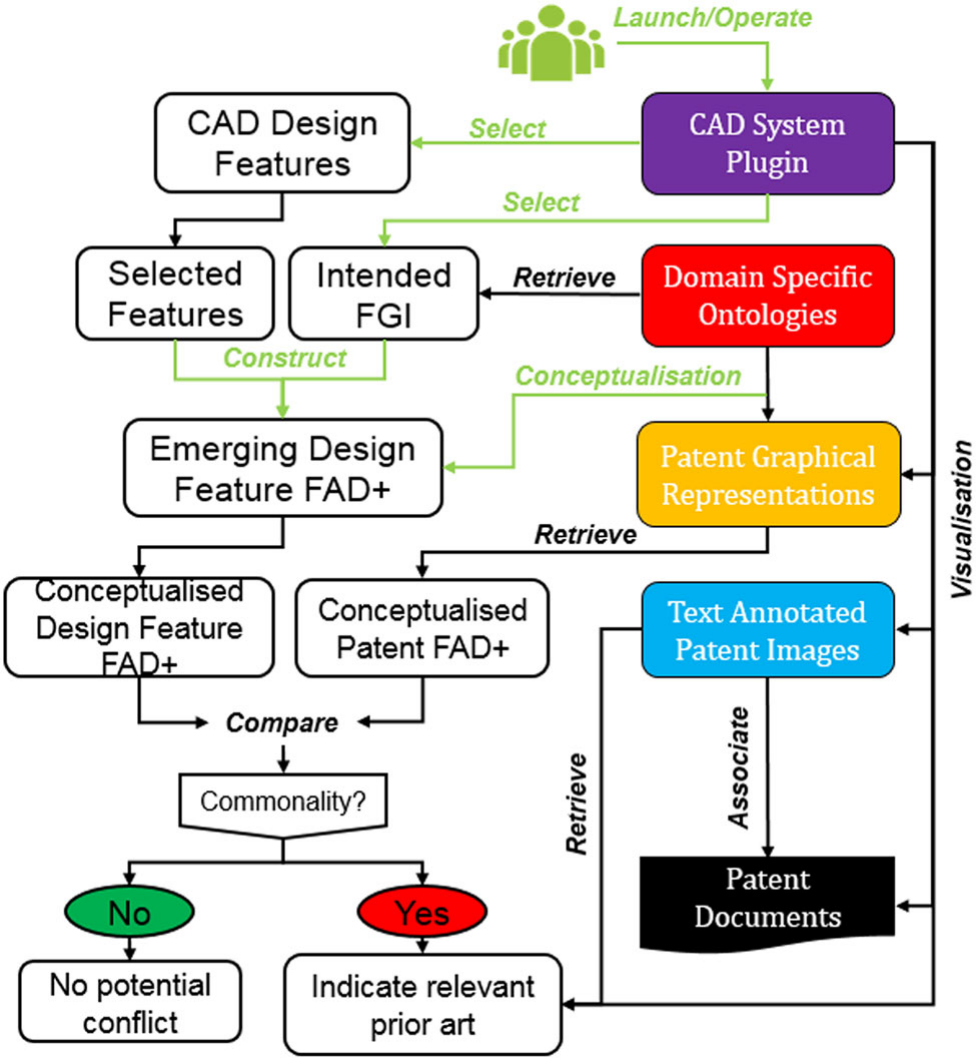
DASCIP in a design process. Green arrows indicate actions required by the designer

Figure 23 illustrates how DASCIP will operate within a design process to check for potential prior art conflict between the emerging design and relevant patents. It is important to note that for existing patents to be identified, they will have been annotated using the methods described in this paper.

A CAD system plugin will allow the designer to operate DASCIP seamlessly within the CAD modelling process. The prior art conflict check starts with the designer selecting the CAD design features he/she wants to check within the CAD system. Domain-Specific Ontologies will provide a list of intended FGI for the designer to choose from in order to construct a FAD+ annotation of the design features. The ontology will be used to perform conceptualisation of design terms and patent terms to enable comparison. If similarity of FGI is identified, then the potential conflicted patents will be retrieved from the database and displayed in the CAD system plugin in the form of annotated patent images. The implications for the designer are that implementing the method described will change their design practice to become more aware of relevant prior art earlier in the design process than with much of current practice. Designer understanding of relevant working principles should improve with more exposure to patented prior art.

## Conclusion

It has been shown how to annotate design images in terms of graphical representation and concise text summaries that capture the working principles of several mechanical designs. Underlying these annotations is a novel functional representation approach that is an expansion of the well-known function analysis diagrams (FAD). This expanded FAD is presented as FAD plus or FAD+ and incorporates a more detailed representation of the interacting geometric features of the devices represented; we have termed them FGI. The working principles and associated FGI contained in several example design cases of differing complexity are addressed through the application of FAD+. The experience suggests that FAD+ is more powerful when analysing inventions that are generally not too complex and where the working principle relies more on interaction between geometric features than between components.

The overall approach described in this paper provides a means of improving awareness of prior art during a design process and thereby suggests stimulating more inventive designs, as well as avoiding conflict with prior art and in turn possibly patent infringement. FAD+ forms the core of a Design Assistant for Comparison of IP (DASCIP) being developed that will be tested on domain experts in can design where geometric features are clearly pivotal to the working principle and has relevance to other domains. The core elements and development process of DASCIP have been briefly discussed together with how it is proposed to fit within a design process.

## References

[CR1] Abbas A, Zhang L, Khan SU (2014). A literature review on the state-of-the-art in patent analysis. World Patent Inf.

[CR2] Altshuller G (1996). And suddenly the inventor appeared: TRIZ, the theory of inventive problem solving.

[CR3] Aurisicchio M, Bracewell R, Armstrong G (2012) The function analysis diagram. In: Proceedings of the ASME 2012 international design engineering technical conferences and computers and information in engineering conference, Chicago

[CR4] Aurisicchio M, Bracewell R, Armstrong G (2013). The function analysis diagram: Intended benefits and coexistence with other functional models. AI Eng Design Anal Manuf.

[CR5] Bhatti N, Hanbury A (2013). Image search in patents: a review. Int J Doc Anal Recogn.

[CR6] Bontcheva K, Tablan V, Cunningham H, Ferro N (2014). Semantic search over documents and ontologies. Bridging between information retrieval and databases.

[CR7] BS8888 (2013) Technical product documentation and specification. https://www.bsigroup.com/en-GB/standards/. Accessed 9 June 2017

[CR8] Camba J, Contero M, Johnson M, Company P (2014). Extended 3D annotations as a new mechanism to explicitly communicate geometric design intent and increase CAD model reusability. Comput Aided Des.

[CR9] Cambria E, White B (2014). Jumping NLP curves: a review of natural language processing research. IEEE Comput Intell Mag.

[CR10] De Nicola A, Missikoff M (2016). A lightweight methodology for rapid ontology engineering. Commun ACM.

[CR11] Devoino IG, Koshevoy OE, Litvin SS, Tsourikov V (1997) Computer-based system for imagining and analysing an engineering object system and indicating values of specific design changes. US Patent 6056428

[CR12] Goldfire Technical Knowledge Discovery (2017) https://www.ihs.com/Info/0216/goldfire-technical-knowledge-discovery.html. Accessed 9 June 2017

[CR13] Guha RV, McCool R, Miller E (2003) Semantic search. In: Proceedings of the 12th WWW conference (WWW 2003), Budapest, pp 700–709

[CR14] Henderson MR (1993) Representing functionality and design intent in product models. In: Proceedings on the second ACM symposium on solid modeling and applications (SMA’93), pp 387–396

[CR15] Hirtz J, Stone R, McAdams D, Szykman S, Wood K (2002). A functional basis for engineering design: reconciling and evolving previous efforts. Res Eng Design.

[CR16] Hubka V (1982). Principles of engineering design.

[CR17] ISO 14405 (2011) Geometrical product specifications. Organisation internationale de normalisation, Genève, Suisse. https://www.bsigroup.com/en-GB/standards/. Accessed 9 June 2017

[CR18] ISO 16792 (2006) Technical product documentation—digital product definition data practices. Organisation internationale de normalisation, Genève, Suisse. https://www.bsigroup.com/en-GB/standards/. Accessed 9 June 2017

[CR19] ISO 5459 (2011) Geometrical product specifications (GPS)—geometrical tolerancing—datums and datum systems. https://www.bsigroup.com/en-GB/standards/. Accessed 9 June 2017

[CR20] Iyer GR, Mills JJ (2006). Design intent in 2D CAD: definition and survey. Comput Aided Design Appl.

[CR21] Jansson DG, Smith SM (1991). Design fixation. Des Stud.

[CR22] Jiang P, Atherton M, Harrison D, Malizia A (2017) Framework of mechanical design knowledge representation for avoiding patent infringement. In: ICED17, 21–25 August 2017

[CR23] Koller R (1973) Eine algorithmisch-physikalisch orieiitierte konstruktioiismethodik (in German). VDI-Z 115:2, 4, 10, 13

[CR24] Lee S, Jiang P, Childs PR (2013) Functional analysis diagrams with the representation of movement transitions. In: ASME 2013 international mechanical engineering congress and exposition, volume 12: systems and design, San Diego, California, USA, November 15–21, 2013, pp V012T13A027, 10 pages

[CR25] Li M, Langbein FC, Martin RR (2010). Detecting design intent in approximate CAD models using symmetry. Comput Aided Des.

[CR26] Li Z, Atherton M, Harrison D (2014). Identifying patent conflicts: TRIZ-led patent mapping. World Patent Inf.

[CR27] Mandorli F, Otto HE, Rafaeli R (2016). Explicit 3D functional dimensioning to support design intent representation and robust model alteration. Comput Aided Design Appl.

[CR28] Michalakoudis I, Childs PR, Aurisicchio M, Pollpeter N, Sambell N (2014) Using functional analysis diagrams as a design tool. In: ASME 2014 international mechanical engineering congress and exposition, pp V011T14A011–V011T14A011. American Society of Mechanical Engineers

[CR29] Pahl G, Beitz W, Wallace K (1988). Engineering design—a systematic approach. The design council.

[CR30] Rodenacker WG (1966). Physikalisch orientierte konstruktionsweise (in German). Konstruktion.

[CR31] Roth K, Frank H-J, Simonek R (1972). Die allgemeine Funktionsstruktur, ein wesentliches hilfsmittel zum methodischen konstruieren (in German). Konstruktion.

[CR32] Salomons OW, van Houten FJ, Kals HJJ (1993). Review of research in feature-based design. J Manuf Syst.

[CR33] Sanfilippo EM, Borgo S (2016). What are features? An ontology-based review of the literature. Comput Aided Des.

[CR34] Ulrich KT, Eppinger SD (2011) Product Design and Development, 5th Edn, McGraw-Hill

[CR35] UK Intellectual Property Office (2014) The Patents Act 1977 (amended):101. https://www.gov.uk/government/publications/the-patents-act-1977. Accessed 9 June 2017

[CR36] UK Intellectual Property Office (2016) Patent factsheets. https://www.gov.uk/government/publications/patent-fact-sheets. Accessed 9 June 2017

[CR37] Verbitsky M (2004) Semantic TRIZ. http://citeseerx.ist.psu.edu/viewdoc/download. Accessed 9 June 2017

[CR38] Vrochidis S, Papadopoulos S, Moumtzidou A, Sidiropoulos P, Pianta E, Kompatsiaris I (2010). Towards content-based patent image retrieval: a framework perspective. World Patent Inf.

[CR39] Vrochidis S, Moumtzidou A, Kompatsiaris I (2012). Concept-based patent image retrieval. World Patent Inf.

[CR40] Weatherall K, Webster E (2014). Patent enforcement: a review of the literature. J Econ Surv.

[CR41] World Intellectual Property Indicators (2014) World Intellectual Property Organisation. http://www.wipo.int/ipstats. Accessed 9 June 2017

